# Production of Bovine Equol-Enriched Milk: A Review

**DOI:** 10.3390/ani11030735

**Published:** 2021-03-08

**Authors:** Ludmila Křížová, Veronika Křešťáková, Kateřina Dadáková, Tomáš Kašparovský

**Affiliations:** 1 Department of Animal Breeding, Animal Nutrition and Biochemistry, Faculty of Veterinary Hygiene and Ecology, University of Veterinary and Pharmaceutical Sciences, 61242 Brno, Czech Republic; krizoval@vfu.cz; 2 Department of Biochemistry, Faculty of Science, Masaryk University, 61137 Brno, Czech Republic; krestakova@mail.muni.cz (V.K.); k.dadakova@mail.muni.cz (K.D.)

**Keywords:** cows, dairy, carry-over, isoflavone, metabolism, health

## Abstract

**Simple Summary:**

Milk and dairy products contain many substances beneficial to human health; moreover, the contents of some of these substances can be enhanced. This is also the case of isoflavones which are compounds of plant origin that can be ingested and metabolized by cattle and, subsequently, secreted into bovine milk. An especially healthful substance called equol is ranked among isoflavone metabolites, commonly produced in the digestive tract of cattle. Equol content in milk can be modified by using feedstuffs with different contents of isoflavones or by milk processing and storage.

**Abstract:**

Milk and dairy products are important sources of nutrients in the human diet because they contain a number of essential substances and other biologically active components. Many of these substances can be modified, and thus offer opportunities to use milk and dairy products as functional food. Isoflavones are particularly important in human nutrition due to their diverse pharmacological and antioxidant properties. The clinical effectiveness of isoflavone-rich products is believed to be dependent on their ability to metabolize daidzein to equol, which may directly exert cancer preventive effects. However, only approximately 30–40% of humans are able to produce equol, while animals, in general, produce equol. Equol is the predominant product of bacterial metabolism of isoflavones and can be found in various amounts in some food of animal origin, especially in milk. Therefore, milk and dairy products can be considered to be sources of equol for humans who are not able to produce this metabolite. When the content of isoflavones in milk is to be modified, two groups of factors should be considered, i.e., dietary factors that include the source of isoflavones and the processing effects on feedstuffs and animal factors that include the intake of isoflavones, ruminal and postruminal changes, and the health and physiological status of animals. The approximate content of isoflavones in milk can be predicted using carry-over rates for different dietary sources or using a formula that describes the relationship between equol concentration in milk and formononetin intake. Processing and storage can affect the content and profile of isoflavones in milk and dairy products.

## 1. Introduction

Functional food includes natural or processed foods that contain unknown or known biologically active compounds [1]. These compounds have a documented health benefit for treatment or prevention of chronic diseases [2]. Milk and dairy products contain a number of essential substances (vitamins, bioactive peptides, highly absorbable calcium, and probiotic bacteria) and other biologically active components [3]. Furthermore, the composition of numerous of these substances can be modified. These properties, together with their prevalence, offer opportunities to use milk and dairy products as functional foods [4]. Isoflavones, which are studied especially in soya products, are among biologically active substances whose content in milk and dairy products can be increased. Nowadays, studies have focused on modifying cows’ feed to produce dairy products enhanced in isoflavones [5].

For the purpose of this review, a literature search was conducted using Web of Science and Science Direct databases. For the section on “Roles of isoflavones in human health, the Web of Science was searched for articles using combinations of search terms “isoflavones” and “menopause”, “cancer”, “antioxida*”, or “*estrogen*”. For the remaining chapters, the search terms included combinations of terms “isoflavones” and “rumen”, “metabolism”, “milk”, “dairy”, or “feed” and were limited to topic/title/keywords. Titles, abstracts, and finally, full manuscripts were manually reviewed for relevance; only articles published in English in peer-reviewed journals and corresponding to the topic of this review were included. Articles published between 1 January 2010 and 1 September 2020 were preferred. Studies that focused on ruminants other than bovine animals or their milk/dairy products were excluded.

## 2. Roles of Isoflavones in Human Health

Isoflavones have been associated with diverse beneficial effects on human health; however, they occur in many chemical forms differing in bioavailability and efficacy. Generally, the most effective are isoflavone aglycones and equol, a metabolite of daidzein [6].

**Figure 1:**
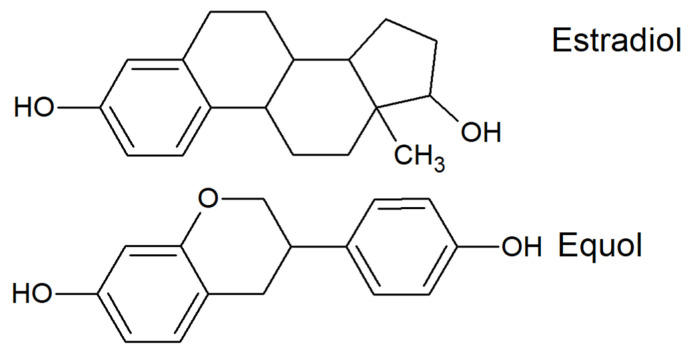
Comparison of estradiol and equol chemical structures.

The most important property of isoflavones, regarding their role in human health, is their structural similarity to oestrogens (Figure 1). Thanks to this similarity, isoflavones and their metabolites are able to bind to oestrogen receptors and activate them, albeit less effectively than physiological oestrogens [7]. The rank order of isoflavones and their metabolites according to their oestrogenic potency is as follows: equol and genistein > glycitein > daidzein > biochanin A and formononetin. They behave as weak oestrogens, and, on the one hand, in the case of oestrogen deficit, they exert a beneficial effect [8], on the other hand, in high concentrations, they act as oestrogen antagonists, blocking the effects of endogenous oestrogens [9]. Thus, they may be considered to be endocrine disruptors and their effects on a certain part of the population may be negative [10,11]. Predicted maximal plasma concentrations after consumption of soy supplement or an Asian diet are 10^3^ and 10^−1^ nM for daidzein and equol, respectively, and after consumption of a Western diet, predicted maximal plasma concentrations are 10^1^ and 10^−2^ nM for daidzein and equol, respectively [12]. The concentrations needed to reach 10% activation of estrogen receptor (ER)-α mediated gene expression as compared with oestradiol activation are approximately 60 and 10 nM for daidzein and equol, respectively [12]. However, while the affinities of oestradiol to the two types of oestrogen receptors, ER-α and ER-β, are approximately equal [13], isoflavones and their metabolites bind to ER-β with greater affinity than to ER-α [14]. Contrary to ER-α binding, binding to ER-β is connected to beneficial antiproliferative activity [15].

Independently from their oestrogen activities, isoflavones also possess antioxidant properties [16]. These effects are due to the hydroxyl groups attached to the aromatic rings. These phenolic groups can be converted to stable radicals during oxidative stress, leading to termination of radical reactions [17]. Human clinical studies that investigated the effects of soy bioactive compounds, including isoflavones, on oxidative stress markers, were recently reviewed by Rizzo [18]. The results were ambiguous. Among eleven studies on isolated isoflavones, ten studies examined the effect of isoflavones on fatty acid oxidative stress; three of the studies found an improvement, one study reported a partial effect, and the other studies did not find any significant effect. Five studies examined the effect of isoflavones on endogenous oxidation markers; three studies found an improvement and two studies did not find any significant effect. In addition, two studies found a reduction of nucleic acid damage.

### 2.1. Health Effects of Isoflavones and Their Metabolites

**Figure 2:**
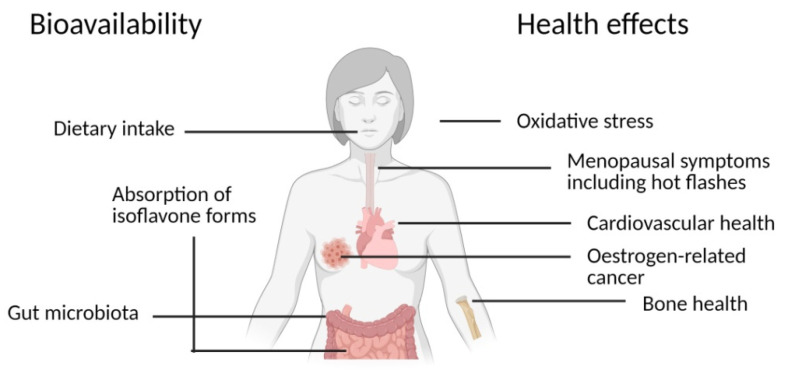
Factors affecting isoflavone bioavailability and isoflavone health effects. Created with BioRender.com.

Menopausal symptoms are connected to oestrogen deficiency and include vasomotor symptoms (primarily hot flashes), urogenital symptoms, metabolic changes leading to weight gain and higher risk of cardiovascular diseases, and accelerated bone loss leading to osteoporosis [19]. Dietary supplements containing isoflavones can help to reduce the menopausal symptoms (Figure 2) including frequency of hot flashes in menopausal women [20], changes in cholesterol forms [21], and the incidence of subclinical cardiovascular disease [22]. However, at least some of the effects were found only in women who were able to metabolize daidzein into equol [19]. Furthermore, genistein was found to suppress osteoporosis. The mechanism of this beneficial effect probably includes vitamin D receptor [23] and genistein was proven to antagonize the catabolic effect of parathormone in osteoblasts, decrease circulating thyroid hormone levels, increase osteoblastic factors, and decrease osteoclastic factors [24]. Apart from genistein, isoflavone aglycones and equol were shown to have a prophylactic effect against bone mass losses [25]. Thus, the impact of isoflavones on bone health may also be dependent on the ability of women to produce equol [19,26,27,28].

Consumption of soy isoflavones is inversely associated with deaths from cardiovascular diseases [29]. Apart from lowering low density lipoprotein (LDL) cholesterol, isoflavones were suggested to have other beneficial effects on cardiovascular health (Figure 2). Soy isoflavones were shown to lower blood pressure in a hypertensive population [30] and in persons whose serum calcium concentration approached lower or upper physiological limits [31], but not in a population with normal blood pressure [30]. Regarding arterial functions, the results were recently reviewed by Man et al. and, predominantly, isoflavones reduced arterial stiffness [32]. Again, equol was reported to be particularly effective for improving arterial stiffness [33]. Isoflavones are able to induce nitric oxide production, and thus prevent endothelial cell dysfunction [34]. Moreover, in a recent study, biochanin A was reported to have a vasodilatory effect in micromolar concentrations by inhibiting the L-type calcium channels [35].

Isoflavones have been suggested to have anticancer properties (Figure 2), primarily in estrogen-related cancer types, such as uterine, breast, and prostate cancer [36]. The incidence of breast and prostate cancer were found to be lower in Asian countries with a higher daily intake of isoflavones than in Western countries [36]. On the one hand, dietary intake of soy isoflavones (genistein and daidzein) was related to a decreased incidence and mortality of prostate cancer [37]. On the other hand, one study reported that an elevated risk of advanced prostate cancer was associated with the dietary intake of isoflavones [38]. Regarding the molecular mechanisms of anticancer effects, the most studied isoflavone is genistein. Genistein is able to alter apoptosis, the cell cycle and angiogenesis, and to inhibit metastasis [36]. Furthermore, chickpea isoflavones (mainly formononetin and biochanin A), as well as equol, were found to induce apoptosis in breast cancer cells [12,39], although plasma concentrations of equol were not found to correlate with breast cancer risk [40].

### 2.2. Metabolism of Isoflavones

The main dietary sources of isoflavones in humans are derived from soybean which contains mainly daidzein and genistein [41] (Figure 2). In soybean, isoflavones are almost exclusively conjugated to sugars, and food processing, primarily fermentation, may lead to their partial deglycosylation [42]. After consumption, they are hydrolyzed mainly in the small intestine by bacterial glucosidases [19] (Figure 2). Contrary to glycosides, the released aglycons are bioavailable and can be absorbed across the intestinal epithelium [43]. Part of the isoflavones, however, passes to the large intestine and can be metabolized by intestinal microflora [43]. Genistein is metabolized to dihydrogenistein and further to p-ethyl-phenol and 6-hydroxy-O-desmethylangolensin. Daidzein is converted to dihydrodaidzein and further to S-equol or O-desmethylangolensin (O-DMA) [44]. Equol has a higher oestrogenic potency, higher antioxidant properties, longer half-life, and is absorbed more efficiently across the intestinal wall than daidzein [19,45] and the biological activity of O-DMA is similar or weaker than that of equol [46].

However, equol is produced only in some human individuals [47], depending solely on the intestinal microflora (Figure 2). About 50% of the Asian population and 70–80% of the Western population do not excrete equol, even if they have consumed soybean products or pure isoflavones [19], because, for a reason that is not known yet, they do not harbor equol-producing bacteria [48]. Several bacterial strains that possess the required enzymes (daidzein reductase, dihydrodaidzein reductase, tetrahydrodaidzein reductase, and dihydrodaidzein racemase) and are involved in the metabolism of daidzein to equol have been identified [49,50]. Even though the list of the identified intestinal bacteria involved in the daidzein metabolism may not be exhaustive, the majority seems to be part of the family *Coriobacteriaceae* [49].

Many studies have proven that, unlike humans, animals commonly produce equol [50]. In ruminants, the metabolites of soybean isoflavones (daidzein, genistein, and glycitein) and red clover isoflavones (formononetin, biochanin A, daidzein, and genistein) include equol, p-ethyl-phenol, and O-DMA [8]. The metabolic conversion of isoflavones takes place mainly in the rumen and the dominant isoflavone found in the digesta of cattle [51] is equol. Relatively large proportions of isoflavones are excreted in milk, and again, the dominant isoflavone is equol [52]. Therefore, milk and dairy products can be considered to be a source of equol for humans not able to produce this metabolite [53,54]. However, for the purposes of functional food production, the feed of dairy cattle, as well as milk processing should be considered, as these parameters substantially influence equol concentrations [53,55,56].

## 3. Update of Isoflavone Occurrence in Bovine Milk and Dairy Products

According to previous results, the isoflavone concentrations in bovine milk range from non-detectable amounts to units (biochanin A and formononetin), tens (daidzein and glycitein), or hundreds of nanograms per milliliter (genistein) [57,58,59]. The concentration of equol is generally higher, ranging from 4 to 1000 ng/mL in raw milk, whereas the dairy processing may influence the content in both directions [53,54,57]. The content of isoflavones in bovine milk and dairy products is likely the result of direct transfer from the feeds, including leguminous plants, which are naturally rich in isoflavones [60]. The most significant source of isoflavones, concerning dairy cattle feeding, is red clover (*Trifolium pratense*), a source of biochanin A, genistein, daidzein, and formononetin [5]. Furthermore, soybean (*Glycine max*) which is a source of daidzein, genistein, and glycitein, is also commonly used as a feed. Even though there are other plants rich in isoflavones, such as kudzu (*Pueraria lobate*) or licorice (*Glycyrrhiza glabra, Glycyrrhiza uralensis*), these plants do not significantly alter the volume of isoflavones in bovine milk and in dairy products [5,61].

Several studies have reported that cows grazing a pasture containing red clover [62], fed isoflavone-enriched feed [57], or red clover/grass silages [63] increased the concentration of isoflavones and especially equol in bovine milk as compared with cows grazing on pasture or feed with other botanical compositions [57,62,63]. The addition of soybean increases the isoflavone levels in milk to a lower extent [5] and the isoflavone concentration in such milk is lower as compared with the soy-based food [64]. In any case, milk produced during the indoor feeding periods contains more equol than milk produced during the outdoor feeding period, because pastures contain less red clover than the fields intended for silage production [65]. Accordingly, milk produced during the summer contains less isoflavones than milk produced during the winter [58]. On the one hand, organic retail milk contains, among other phytoestrogens, higher levels of isoflavones than milk originating from conventional or free-range systems. On the other hand, the isoflavone levels do not differ between free-range and conventional milk [58,65]. In conclusion, the isoflavone-enriched feed does not have a positive effect on the milk production [57].

## 4. Factors Affecting the Content of Isoflavones in Bovine Milk

**Figure 3:**
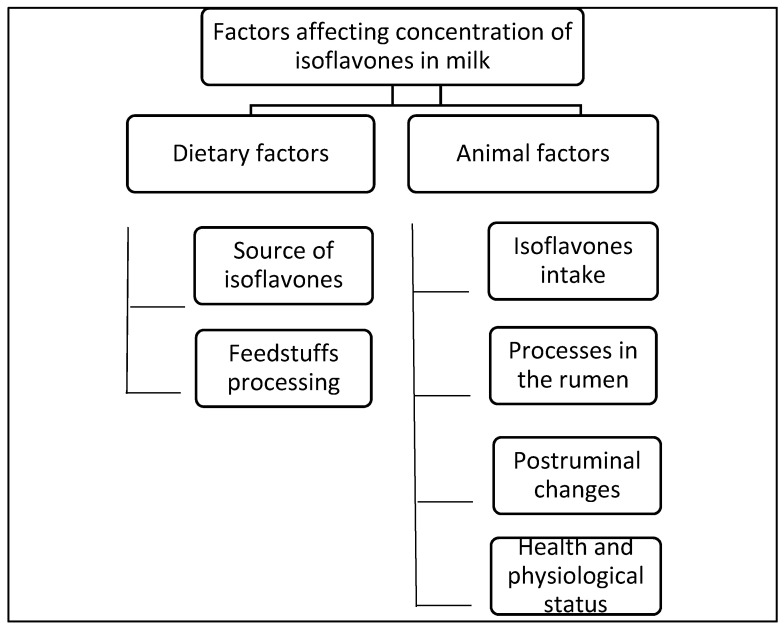
Factors affecting the concentration of isoflavones in milk.

Factors that should be taken into consideration when the content of isoflavones is to be manipulated are summarized in Figure 3.

### 4.1. Dietary Factors

#### 4.1.1. Source of Isoflavones

As reviewed recently in [5], red clover-derived or soybean-derived feedstuffs are relevant sources of isoflavones in dairy diets. However, isoflavones also occur in other feedstuffs used for feeding dairy cattle, such as white clover (*Trifolium repens*) and other clover species [66], lucerne (*Medicago sativa*) [67] and birdsfoot trefoil (*Lotus corniculatus*) [68]. However, concentrations of isoflavones in those feedstuffs are considerably lower than in red clover.

In both, red clover and soybean, the content of isoflavones is significantly influenced by variety, planting location, and year or applied fertilization [69,70,71,72].

In red clover, isoflavones are distributed unevenly in aboveground parts and their concentrations depend on the phenological stage, leaf/stem ratio, and the season of the plant [65]. Generally, the highest isoflavone contents were reported in leaves, followed by stems, petioles, and flowers, with formononetin and biochanin A being the predominant isoflavones in all parts [73]. The highest concentrations of formononetin (51%) and biochanin A (40%) were accumulated in leaves during the flowering stage [73]. The concentration ranges of formononetin, biochanin A, daidzein, and genistein at the flowering stage were 2.61–4.40, 1.79–3.32, 0.06–0.14, and 0.36–0.59 mg/g dry matter (DM), respectively. The average total concentration of all four isoflavones at the flowering stage were as follows: 12.29 mg/g DM in leaves, 2.93 mg/g DM in stems, and 1.42 mg/g DM in flowers [73].

In soybeans, the concentration of isoflavones correlated with protein content in seeds [74].

#### 4.1.2. Processing of Feedstuffs

The processing methods applied on isoflavone-rich feedstuffs differ. Generally, forages such as clovers are processed mainly by preserving techniques, either drying (haymaking) or ensiling. For grains such as soybeans, we can use a whole range of mechanical, (hydro) thermal, or chemical treatments. However, the information about the effect of processing on isoflavones content is incomplete because the concentration of isoflavones in final products (e.g., in red clover silage or soybean meal) is usually reported in the literature.

In the case of forages, field-drying of red clover fresh material decreased formononetin and total isoflavones contents by 28 and 22%, respectively, while there was no effect on the concentration of biochanin A was observed [75]. Decreases in formononetin and daidzein contents by about 13 and 7–15%, respectively, were also observed during wilting prior to ensiling [76]. However, Daems et al. [77] found no effect of wilting on isoflavones, except for daidzein that increased twice during the four-day wilting. The effects of ensiling on isoflavones were inconsistent. While Sarelli et al. [76] found about 18% higher concentration of isoflavones in ensiled red clover as compared with wilted or fresh material, Sivesind and Seguin [75] described a decrease in formononetin and total isoflavones content by 20 and 22%, respectively, and no effect of ensiling on biochanin A. A recent study performed in laboratory micro-silos revealed that the isoflavone concentrations drastically decreased within the first two weeks of fermentation, where losses of formononetin and biochanin A were 73% and 66%, respectively, but then the isoflavone concentration was stable for the remaining 5.5 months [77]. However, it should be noted that ensiling is a complex and dynamic process and can be influenced by many factors, among others, by the quality of ensiled forage, pre-ensiling processes, or usage of silage additives [78] and these factors, either individually or in combination, can influence isoflavone concentrations in the preserved material. Further studies are needed to clarify the effect of various ensiling conditions on isoflavones.

In the case of soybean feedstuffs, changes in isoflavones during processing were not systematically studied but some information was applicable from the food industry, although these studies were often focused on interconversion changes between various isoflavones types and alteration in isoflavone profile (e.g., [79]). Solvent-extracted soybean meal is the most common by-product of the oil industry intended for dairy feeding. During the processing, pre-extraction treatments include cracking, dehulling (optional), heating and flaking, and post-extraction treatments include drying, toasting, and grinding. The extraction is done by hexane [80]. Concerning the heat treatment, pasteurization or heating to 140 °C for 20 s had no effect on isoflavones content [81]. Even though hexane extraction does not alter the composition of isoflavones [79], solubility of individual isoflavones in hexane differs, being higher for genistein as compared with daidzein [82].

### 4.2. Animal Factors

Daily intake of isoflavones depends on the dietary source of isoflavones. As mentioned above, we consider two main isoflavones sources in dairy diets, i.e., red clover and soybean which differ in isoflavone content; the content of isoflavones is higher in red clover than in soybean, i.e., 0.8–11 mg/g and 1.2–4.2 mg/g dry weight, respectively [73,83]. Moreover, red clover is a bulky feed and can represent up to 97% of the dry matter intake. On the other hand, usage of soybean and soybean products in dairy diets is limited because soybeans, as concentrated feedstuffs, are high in protein and also in fat if used in the form of (treated) full-fat products. The maximum inclusion rate reported in the literature was up to 30% of the total diet dry matter [64]. Thus, logically, higher isoflavone intakes are achieved from red clover-based diets (see Table 1). However, we can avoid this limitation by using soybean extracts that can significantly increase the daily isoflavones intake without an imbalance of main nutrients in the diet [57].

Dietary isoflavones are extensively metabolized in the rumen by ruminal microorganisms [51]. Their half-lives in the bovine rumen fluid are 4.3 h for formononetin, 9.3 h for daidzein, 3.9 h for biochanin A, and 5.5 h for genistein [84] and their metabolites occur after 3 h of incubation in the rumen fluid in vitro [55,56]. Concerning the main metabolites, formononetin is demethylated to daidzein, and then further metabolized to equol, a compound with significant estrogenic activity, while biochanin A is demethylated to genistein and further converted to p-ethyl-phenol with no estrogenic activity [5]. The course of metabolism in the rumen can be influenced by the type of diet and by the concentration of isoflavones in the diet [55,56]. Moreover, interaction between isoflavones and rumen bacteria should be also considered, as stimulatory/inhibitory effects of isoflavones on growth of some rumen bacteria were recently reported [85,86,87].

Although the majority of dietary isoflavones is degraded or absorbed in the rumen, small proportions of unmetabolized isoflavones, in concrete 0.4–8% of biochanin A and genistein and 7–15% of formononetin, daidzein, and their metabolite equol were found in omasum [51]. The fate of unmetabolized isoflavones in the intestine of ruminants has not yet been thoroughly described. We suppose that the processes involving metabolism by intestinal bacteria, the conjugation and sulphatation in the gastrointestinal tract, and the re-conjugation in liver are similar as described in human or other animal species, for example, rats [92]. The capacity of epithelial tissue for those reactions in the gastrointestinal tract probably limits the carry-over rate of isoflavones from feed into milk [51].

There are limited studies that have focused on the effect of animal health and physiological status on metabolism of isoflavones. Kowalczyk-Zieba et al. [93] found differences in isoflavone absorption and isoflavone metabolite levels in the blood plasma of cows with experimentally induced mastitis and metritis. These differences were caused by a higher activation of β-glucuronidase during mobilization of the immune system of affected cows. Furthermore, differences in the immunological status of cows connected with the phase of pregnancy can also affect the course of isoflavone metabolism because early pregnant animals had a higher concentration of metabolites in plasma as compared with animals in late pregnancy [94].

## 5. Prediction of Isoflavones Content in Milk

The quantitative aspect of isoflavone transfer from feed into milk is commonly expressed as the carry-over rate or apparent recovery [88] and can be calculated for individual as well as total isoflavones. Generally, it is calculated as a ratio between isoflavones secreted in milk and isoflavone intake. From the view of human health, the transfer of daidzein (or daidzein + formononentin) and their metabolite equol into milk is the most important, therefore, their carry-over rate is calculated as follows:

$$carry\;over\;rate\;\left(\mu g/mg\right)=\frac{\sum daidzein\;\left(+formononetin\right)+equol\;secreted\;in\;milk\;\left(\mu g/d\right)}{\sum daidzein\;\left(+formononetin\right)\;intake\;\left(mg/d\right)}$$

Carry-over rates reported in the literature differ in dependence on the isoflavone source and their daily intake, as documented in the Table 1. However, they can be considered to be a tool for an approximate prediction of isoflavone concentration in milk or for modification of diets for production of desirable amount of isoflavones in milk. Another tool available for prediction purposes is the relationship between equol concentration in milk and formononetin intake, i.e., y = 0.0035x + 0.358, published by Mustonen et al. [95], but the relationship is weak (R^2^ = 0.20). No relationship is available for soybean-derived isoflavones. Further studies are needed to improve our knowledge of the quantitative aspects of isoflavone transfer from feed into bovine milk.

## 6. Changes in Isoflavone Contents during Technological Processing of Milk and Dairy Products

**Figure 4:**
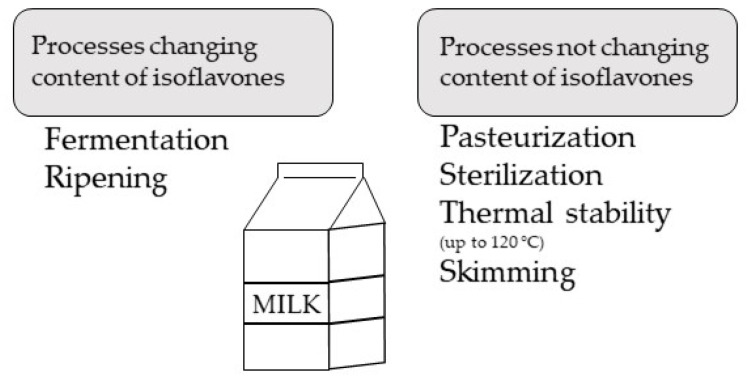
Technological processing affecting concentration of isoflavones in milk.

Recent data have suggested that the concentration of isoflavones in milk and dairy products can be changed by a number of processes during the course of technological processing (Figure 4) [91].

The most common processes, namely sterilization and pasteurization, do not change the isoflavone level, as thermal stability of isoflavones is up to 120 °C [54,91,96]; similarly, skimming does not seem to alter isoflavone concentration [91]. However, raw milk contains a lower concentration of equol than skimmed milk, which implicates a higher affinity of equol for an aqueous fraction [54]. The processes that are involved in the preparation of yogurts do not affect the quantity of isoflavones [54], although maturation, which is the process that follows the preparation of yogurt, has a minor effect on isoflavone content. The glycitein concentration decreases, whereas the concentrations of daidzein, genistein, and equol remain unaffected [53]. Yogurts obtained from isoflavone-enriched milk contain higher concentrations of daidzein and equol, but the levels of genistein and glycitein remain unchanged [53].

In addition, fermentation causes substantial changes in isoflavone content, primarily in the concentrations of daidzein and glycitein. In contrast to fermentation of soymilk, which leads to increased concentrations of isoflavone aglycones, fermentation and ripening of cows’ milk causes high losses of isoflavones [53,96,97]. Similarly, storage decreases the amounts of isoflavones [96]. A study by Otieno et al. [98] showed a smaller degradation of aglycones than glucosides of isoflavones, but only for soymilk. Accordingly, cheese can contain isoflavones, but the concentrations decrease during the ripening process [96]. Low amounts of isoflavones were found in cream and kefir and, surprisingly, isoflavones were detected in whey [54,91].

## 7. Conclusions

Isoflavones in human nutrition are particularly important due to their wide range of pharmacological and antioxidant properties. However, the clinical effectiveness of isoflavones depends on the ability to metabolize daidzein to equol, which is true only for approximately 30–40% of humans. Thus, alternative dietary sources of equol in human nutrition are desirable. From the dietary sources, equol-enriched bovine milk seems to be the most suitable product for individuals lacking the ability to produce equol from its dietary precursors. This review showed that for the production of equol-enriched milk, it is important to know the quantitative aspects of isoflavone transfer from feed into milk and to study factors that can influence this transfer. Improved understanding in postruminal metabolic processes is needed as they were identified as factors affecting the carry-over rates; furthermore, not all aspects of rumen metabolism are clear.

## Figures and Tables

**Table 1:** The carry-over rates of daidzein (+formononetin) from feed into bovine milk from selected dietary sources in relation with the isoflavones intake.

Source of Isoflavones	Intake of Daidzein (+Formononetin) (mg/d)	Carry-Over Rate ^1^ (μg/mg)	Source
Red clover/grass silage	48,410	0.21	[88]
Red clover/grass silage + concentrate	38,700	0.24	[88]
Red clover grass silage, 2nd cut	40,350	1.07	[63]
Red clover grass silage, 3rd cut	39,950	0.96	[63]
White clover/grass silage	2249	1.20	[88]
White clover/grass silage + concentrate	2106	0.92	[88]
Birdsfoot Trefoil grass silage	2520	1.83	[63]
Grass/clover silage	not given	1.23	[89]
Lucerne silage	not given	0.77	[89]
40% Soybean isoflavone extract	10,618	0.50	[57]
Extruded full-fat soybean	1319	1.3	[90]
Extruded full-fat soybean	439	2.5	[91]
Soybean meal	not given	2.0–4.4 ^2^	[64]

^1^, calculated as (sum of equol, formononetin, and daidzein secreted in milk)/(sum of formononetin and daidzein intake). ^2^, The mean total carry-over rate of isoflavones declined with increasing isoflavones intake according to the formula y = −0.0001x + 0.0006 (R^2^ = 0.69).

## Data Availability

No new data were created or analyzed in this study. Data sharing is not applicable to this article.
